# Supplementary material for: Nephrotoxic Metal Mixtures and Preadolescent Kidney Function
Source: Children (Basel). 2021 Aug 2;8(8):673. doi: 10.3390/children8080673 (PMC8391795; doi:10.3390/children8080673)
Supplement: Supplementary file 1 [file children-08-00673-s001.zip › children-1294016-supplementary.pdf]

Supplemental Information

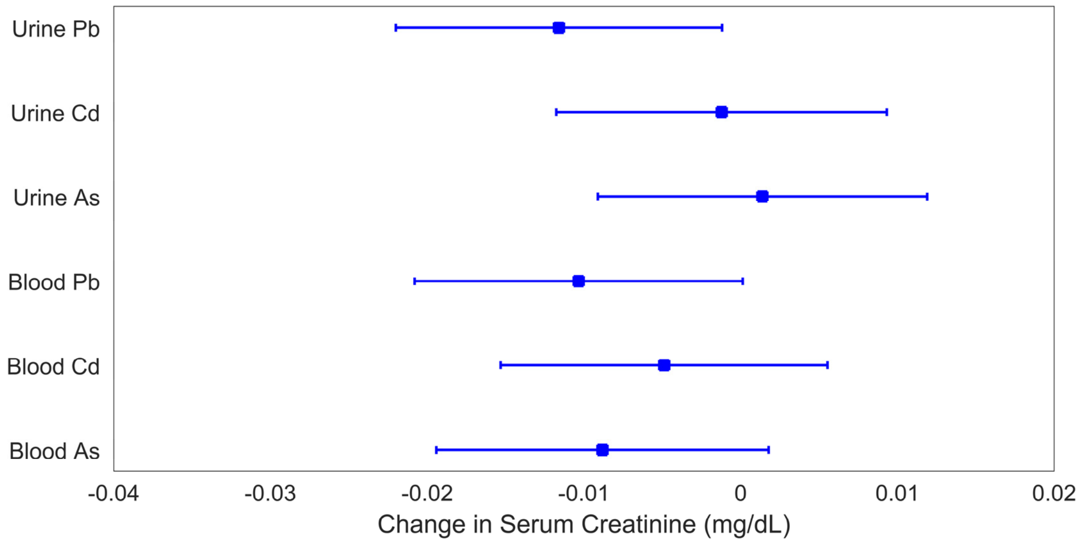

Figure S1: Change in serum creatinine (mg/dL) for a 1-quartile increase in metals levels. Plotted points are beta coefficients and lines are 95% confidence intervals. All models were adjusted for child age (years), child sex, indoor tobacco smoke exposure (referent group: no), SES (referent group: lower), and BMI (referent group: BMI z-score<1). The p-values for these associations are: 0.03 (urine Pb), 0.82 (urine Cd), 0.80 (urine As), 0.05 (blood Pb), 0.36 (blood Cd), 0.10 (blood As).

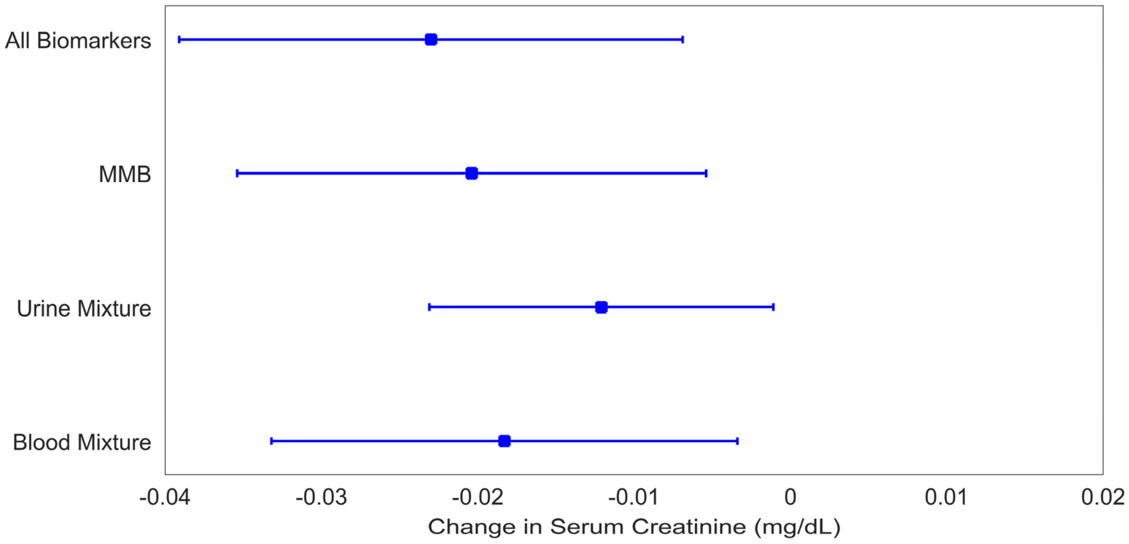

Figure S2: Change in serum creatinine (mg/dL) for a 1-quartile increase in metal levels. Plotted points are beta coefficients and lines are 95% confidence intervals. All models were adjusted for child age (years), child sex, indoor tobacco smoke exposure (referent group: no), SES (referent group: lower), and BMI (referent group: BMI z-score<1). The p-values for these associations are: 0.005 (all biomarkers), 0.008 (MMB), 0.03 (urine mixture), and 0.02 (blood mixture).

Table S1: Weights highlighting the contribution of each metal to the mixture effect of metals on serum creatinine. Larger weights indicated higher contributions to the mixture effect.

	Blood Mixture (B_{mix})	Urine Mixture (U_{mix})	MMB	All Biomarkers
As	39%	9%	35%	30%
Cd	18%	2%	17%	16%
Pb	44%	88%	48%	54%

Table S2: Weights highlighting the contribution of each biomarker to the mixture effect of metals on serum creatinine. Larger weights indicated higher contributions to the mixture effect.

	As MMB	Cd MMB	Pb MMB	MMB Overall	All Biomarkers
Blood	97%	73%	42%	67%	62%
Urine	3%	27%	58%	33%	38%
